# Pathways from Food Consumption Score to Cardiovascular Disease: A Seven-Year Follow-Up Study of Indonesian Adults

**DOI:** 10.3390/ijerph15081567

**Published:** 2018-07-24

**Authors:** Emyr Reisha Isaura, Yang-Ching Chen, Shwu-Huey Yang

**Affiliations:** 1 School of Nutrition and Health Sciences, College of Nutrition, Taipei Medical University, Taipei 11031, Taiwan; d507103003@tmu.edu.tw (E.R.I.); melisa26@gmail.com (Y.-C.C.); 2 Department of Family Medicine, Taipei Medical University Hospital, Taipei 11031, Taiwan; 3 School of Medicine, College of Medicine, Taipei Medical University, Taipei 11031, Taiwan; 4 Nutrition Research Center, Taipei Medical University Hospital, Taipei 11031, Taiwan; 5 Research Center of Geriatric Nutrition, College of Nutrition, Taipei Medical University, Taipei 11031, Taiwan

**Keywords:** food consumption score, body shape index, blood pressures, physical activity, cardiovascular disease, generalised estimating equations, structural equation modelling

## Abstract

*Background*: Available prospective studies of food insecurity and cardiovascular diseases (CVD) have included obesity and hypertension as the modifiable risk factors. Studies using the physical activity measures are lacking, and where to contribute to counterbalance the risk associated with food insecurity and CVD remains unclear. We aimed to use structural equation modelling (SEM) to explore the complex direct and indirect factor variables influencing cardiovascular disease (CVD) during a seven-year follow-up study. *Methods*: For 3955 adults who participated in the Indonesian Family Life Surveys in 2007 and 2014, we used SEM to examine the direct and indirect relationships of food consumption score, body shape index, physical activity volume, and blood pressures on CVD. *Results*: Based on the beta coefficients from a regression analysis, the significant direct effects (*p* < 0.001) for CVD were food consumption score (FCS), a body shape index (ABSI), vigorous physical activity volume (VPAV), and systolic blood pressure (SBP). Indirect (*p* = 0.004–*p* < 0.001) effects for CVD were FCS, ABSI, moderate physical activity volume (MPAV), and VPAV. Food-insecure people are more likely to consume high-calorie diets that lead to obesity, which, together with a lack of vigorous physical activity, leads to hypertension and CVD. *Conclusions*: Of the multiple factors influencing CVD, the modifiable risk factors were FCS, ABSI, and VPAV. Hence, the recommendations for CVD prevention should include targeting food insecurity, body shape index, and vigorous physical activity besides the measurement of blood pressure.

## 1. Introduction

Complex correlations between food insecurity, obesity, hypertension, and cardiovascular diseases (CVD) have been reported [1,2,3]. Food insecurity is defined as an individual’s inability to consume a more diverse and better-quality diet [4,5,6]. Among the several methods of food security measurements, the United World Food Programme (WFP) emphasises the measurement concept of food frequency and diet diversity [7]. Further, the inability to have a balanced meal for a healthy life, worsened by the lack of physical activity, increased their CVD risk factors (i.e., obesity and hypertension) [1]. Available prospective studies of food insecurity and cardiovascular diseases have included obesity and hypertension as the modifiable risk factors. However, the former studies reported that food insecurity is not associated with physical activity [8,9,10]; physical activity together with diet are known as the basic factor of obesity. One indicator of obesity, namely a body shape index (ABSI), are partly mediating the relationship pathways between food insecurity and chronic disease (e.g., hypertension, diabetes, and CVD) [2,11]. Seligman et al. highlighted the complexity of the pathways which are interconnected with the possibility of inducing malnutrition among adults in developing countries, as shown in the cycle of food insecurity and chronic disease [12]. In developing countries such as Indonesia, there are complex pathways where malnutrition among adults is characterised by a coexistent impairment of CVD incidences. One explanation for this phenomenon is the lacking report of the physical activity measurements on the relationship between food insecurity and CVD in developing countries. Mathiassen and Hollema suggested to take physical activity into account for determining the prevalence of food insecurity in developing countries [13]. Several studies have found that a higher volume of physical activity has been associated with a decrease in CVD risk factors [14,15,16,17]. Some cross-sectional studies have shown that physical activity can be considered to be a mediator of poor health outcomes rather than to be a confounder in conceptual models of food insecurity [9,10]. However, where physical activity can counterbalance the risk associated with food insecurity and CVD remains unclear. Thus, this study aimed to find the association among a body shape index, physical activity, hypertension, and the risk of CVD among Indonesian adults.

## 2. Materials and Methods

### 2.1. Data Collections and Participants

This study used data from the Indonesian Family Life Surveys (IFLS), an ongoing longitudinal study started in 1993 [18]. This dataset is the available de-identified dataset for researchers who fulfil RAND Corporation guidelines. The IFLS study followed the institutional review board (IRB) regulations for IRB reviews that it was sufficient and properly reviewed, and it was approved by the RAND Corporation and institutions in Indonesia [19]. We analysed data from subjects who participated in the fourth and fifth wave of IFLS, for a total of seven years of follow-up study. Adult participants aged 18–65 years who completed the food frequency questionnaire (FFQ), and the physical activity (PA) and sociodemographic record, as well as the information on blood pressure were included in the study. In 2014, 8613 (34.8%) of the total participants were completed the FFQ and Physical activity questionnaires of the fourth and fifth wave of IFLS. For the purpose of this study, we expected to explore factor that influencing CVD, so we used participants who was not having any chronic disease. This study included participants without missing data for anthropometric measurements (i.e., stature, weight, and waist circumferences), who were not pregnant or breastfeeding, who were not currently using hypertension or diabetes medication, and who had never been diagnosed as having cancer, cardiovascular disease, or diabetes before the year 2009. Finally, we included the data of 3955 participants (16.0% of total 24,744 adult participants in IFLS 2014 participants) in this study.

### 2.2. Outcome Definitions

We used cardiovascular disease incidents from a combination of heart disease incidents and stroke events [20]. The outcomes were self-reported; the question defining the outcomes was: *Have any doctors/paramedics/nurses/midwives ever told you that you had (…)?* These included: (1) heart diseases: heart attack, coronary heart disease, angina, or other heart problems; and (2) stroke. If a participant reported having any of the outcomes, then they were asked: *When was the condition (…) first diagnosed?* These included the year and/or the age of their first time being diagnosed. We excluded participants who had been diagnosed with any heart diseases before the year 2009.

### 2.3. Blood Pressures and Physical Activity

A participant is said to have hypertension if their systolic blood pressure (SBP) ≥140 mmHg or diastolic blood pressure (DBP) ≥90 mmHg [21]. We used the continuous data of blood pressure (BP) values in the descriptive and correlation analysis. Two self-reported questions assessed physical activity volume with a unit of the metabolic equivalent of one task-hour per week (MET h/w) [22]. Participants were asked whether they engage in vigorous, moderate or walking activities: *During the last 7 days, did you do any (…) for at least 10 min continuously?* If a participant reported engaging in any of the moderate or vigorous PA, then they were asked further: *During the last 7 days, on how many days did you do (…)?* These continuous data on the days when participants engaged in physical activity were then multiplied by the assigned intensity value (i.e., 4 and 8 METs for moderate and vigorous intensity, respectively). Thus, we established the vigorous physical activity volume (VPAV) and moderate physical activity volume (MPAV) as continuous data.

### 2.4. Obesity Measures

A body shape index (in m^11/6^ kg^−2/3^) was calculated from the waist circumference (in centimetres) divided by a multiplication of two-thirds squared of the body mass index (in kg/m^2^) by one-half squared of the stature (in centimetres) [23,24,25]. We categorised participants as having abdominal obesity if their waist circumference (WC) >90 cm for men and >80 cm for women. Moreover, we calculated body mass index (BMI) using kilograms of body weight divided by meters squared of stature [26]. The body mass index of participants was categorised into non-overweight if their BMI value was <25 kg/m^2^ and overweight if their BMI value was ≥25.1 kg/m^2^ [26].

### 2.5. Food Consumption Score and Sociodemographic Characteristics

The current study employed the development of the WFP food security assessment, named the food consumption score (FCS), involving the diversity of diet and food frequency in the food consumption analysis [7,27]. The food consumption analysis was built from the face-to-face interview of the IFLS4 and IFLS5 food frequency questionnaire (FFQ). The FFQ comprised ten food items, including sweet potato, eggs, fish, meat, carrot, green leafy vegetables, mango, papaya, banana, and dairy products [11]. We categorised the FCS continuous data into three food consumption groups (FCG) which further formed the food security (FS) level. The three FCG were “poor” (FCS value < 21), “borderline” (FCS value 21–35), and “acceptable” (FCS value > 35). Further, the FS levels were “food insecure” (poor and borderline FCG) and “food secure” (acceptable FCG) [27]. We performed the descriptive and correlation analyses using continuous data from the FCS.

We used categorical data for sociodemographic characteristics (i.e., smoking status, overweight, and education level) in support of the purpose of this study. We categorised the smoking status of participants into three categories (i.e., never: they never had a smoking habit; current: they currently have a smoking habit; or former: they formerly had a smoking habit). We also further categorised this into two categories (i.e., “yes” if they currently have or formerly had a smoking habit, and “no” if they never had a smoking habit). The education level of participants was categorised into low if they had <12 years of school attainment and high if they had ≥12 years of school attainment.

### 2.6. Statistical Analysis

**Figure 1:**
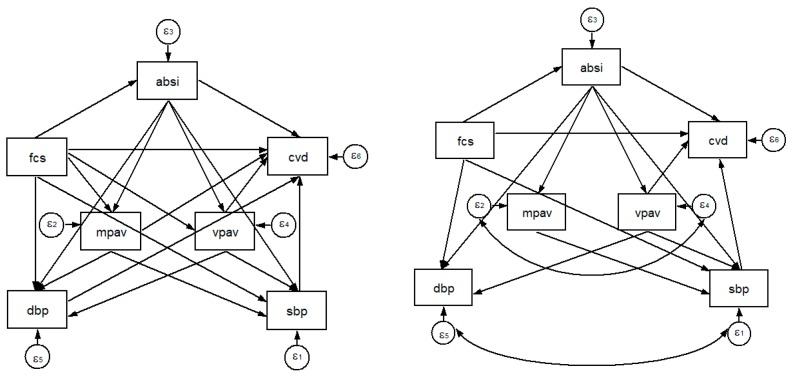
The hypothesised model (**left**) and final model (**right**) of the structural equation modelling (SEM).

We presented the participants’ characteristics as the mean and standard deviation (SD) for the normally distributed continuous variables or as a number and percentage for the categorical variables. A *t*-test was used to compare the values between 2007 and 2014. Based on former works of literature, a pairwise correlation test was used to produce the correlation matrix for each of the variables known or hypothesised to influence the CVD of the adult [28]. General estimating equation (GEE) test is a development of the generalised linear model to observe the association between independent variables and dependent variables [29,30]. GEE test is used for the repeated measurement data or in the longitudinal data analysis. Further, the GEE test was used the Gaussian in the distribution of the dependent variable (family), the identity as the link function, and an independent correlation matrix. This study carried out an independent correlation matrix in the GEE test for the repeated measurements data that have time-dependent. Moreover, we used a generalised estimating equation (GEE) test to observe the association between the physical activity and food consumption score. Structural equation modelling (SEM) is an extension of several multivariate techniques that can examine a series of dependence relationship simultaneously. In particular, to test theories that contain multiple equations involving dependent relationships [31,32]. SEM of food consumption score, body shape index, blood pressures were used to describe the food insecurity, obesity, and hypertension variables influencing CVD. To understand the direct and indirect effects, we used the graphic that shows the hypothesised model using arrows. Analyses proceeded in two stages. First, in agreement with our hypotheses, we created and confirmed the a priori factor structure. We confirmed the first dimension measured in our hypothetical model was a body shape index as the mediator variable between the food consumption score and hypertension. The food consumption score was associated, and mediated some part, with the body shape index, and hypertension (i.e., SBP, DBP) [1,2,3]. An assumption of this analysis is that an underlying unmeasured variable is identified by the shared variance of the observed variables. The sets of factors that comprise physical activity volume, food consumption score, body shape index, and blood pressure may best be modelled in terms of their shared variance rather than to the individual account. Second, we tested the hypothesised model with special emphasis on observing the correlation and estimation of direct effects that define proposed variables. The aim of our hypothesised model was to assess modifiable factors such as food consumption score, body shape index, physical activity and blood pressure that influence CVD. The hypothesised model is shown in Figure 1. This hypothesis model has 19 direct arrows.

The hypothesised model shows each path’s correlation between the proposed variables and CVD, which was investigated using structural equation modelling. We carried out three steps to proceed with the SEM in this study. First, we used the maximum likelihood with missing values (MLMV) method to estimate the variables. Second, we tested the direct and indirect effects, and provided these by using beta coefficients (β). Third, we tested the overall goodness of fit. The fit test includes values from the root mean square error of approximation (RMSEA) of <0.08, from the standardised root mean squared residual (SRMR) of <0.05, from the comparative fit index (CFI), and from the Tucker–Lewis index (TLI) of >0.90 which reflect a good model fit. Statistical significance was inferred at *p* < 0.05. We reached our final model by using the above criteria to reach the best model fitness. This study was conducted using STATA 12.1 (StataCorp LP, College Station, TX, USA) for all analyses.

## 3. Results

### 3.1. Characteristics of Study Participants

Table 1 shows the characteristics of the study participants. The mean age of the participants was 54(5) years of age in 2014, and 51.98% of total participants were women. There were no significant differences between 2007 and 2014 in terms of participants’ education level and marital status. There was a significant difference in the percentage of participants with a smoking habit from 2007 to 2014. The number of participants who did not have a smoking habit in 2007 was 2482 (62.76%), and this number decreased to 2382 (60.23%) in 2014. The number of participants who had a smoking habit in 2007 was 1356 (34.29%), and this number decreased to 1291 (32.64%) in 2014. In 2007, the number of participants who had quit a smoking habit was 117 (2.96%) and this number increased to 282 (7.31%) in 2014.

### 3.2. Assessment of Food Consumption Score and Obesity

The mean food consumption score (FCS) of participants was significantly decreased from 52.02 (22.76) in 2007 to 34.81 (15.77) in 2014. In 2007, 330 (8.34%) of total participants were in the poor FCG, and the numbers significantly increased to 815 (20.61%) during the study period. The percentage of borderline FCG participants increased from 16.43% in 2007 to 32.31% in 2014. The number of the acceptable FCG participants was 2975 (75.22%) in 2007 and decreased to 1862 (47.08%) in 2014 (*p* < 0.001).

Participants who had abdominal obesity increased from 1627 (41.14%) in 2007 to 2080 (52.59%) in 2014. The percentage of participants who were overweight increased from 34.89% in 2007 to 41.24% in 2014. The mean body mass index (BMI) of participants significantly increased from 23.95 (3.96) kg/m^2^ in 2007 to 24.57 (3.90) kg/m^2^ in 2014. The mean waist circumference of participants increased from 82.60 (10.23) cm in 2007 to 86.11 (10.75) cm in 2014 (*p* < 0.001). The mean body shape index (ABSI) of participants was 0.0801 (0.0062) m^11/6^ kg^−2/3^ in 2007, and the number significantly increased to 0.0821 (0.0056) m^11/6^ kg^−2/3^ in 2014 (*p* < 0.001).

### 3.3. Assessment of Physical Activity, Blood Pressure, and Health Outcome

Participants self-reported their physical activity intensity, duration, and frequency, which were then formulated as the physical activity volume (PAV) in MET h/w. The mean of the vigorous physical activity volume (VPAV) of participants decreased from 37.22 (82.47) MET h/w in 2007 to 22.02 (65.63) MET h/w in 2014 (*p* < 0.001). The mean of the moderate physical activity volume (MPAV) of participants was 36.47 (48.80) MET h/w in 2007, and the number significantly decreased to 20.19 (38.82) MET h/w in 2014.

The mean of the systolic blood pressure (SBP) increased from 135 (21) mmHg in 2007 to 143 (25) mmHg in 2014 (*p* < 0.001). The mean of the diastolic blood pressure (DBP) increased significantly from 83 (12) mmHg in 2007 to 85 (14) mmHg in 2014. The prevalence of hypertension among participants increased significantly from 39.52% to 51.83% (*p* < 0.001). Cardiovascular disease incidence was found in 114 of the total participants while diabetes was found in 166 of the total participants by the end of the study period.

### 3.4. Correlation of the Proposed Variables for the SEM

Table 2 shows a correlation matrix of the proposed variables in a pairwise correlation test. The food consumption score represents a food security indicator known to affect chronic disease [33]. A body shape index, physical activity, and blood pressure were included because these have been linked with cardiovascular disease in adults [20]. The VPAV was not significantly correlated with FCS, which in further analyses in GEE testing showed significance after some adjustment.

Table 3 shows the generalised estimating equation (GEE) test scores among food consumption score, blood pressure, and physical activity. Food consumption scores were significantly negatively correlated with SBP, DBP, and VPAV. The significant and negative correlations between FCS and blood pressures (i.e., SBP and DBP) were consistent in all model of the GEE test (*p* = 0.003–*p* < 0.001). The correlation between food consumption score and moderate physical activity is positive (*p* = 0.041–*p* < 0.001). The significant and positive correlation between FCS and MPAV were consistent in the unadjusted and adjusted model of the GEE test. Food consumption score was negatively correlated with the vigorous physical activity volume only if adjusted for age and sex (*p* = 0.008). The meaning is the increase one unit of FCS in the same age and sex group will decrease the VPAV by 0.10 MET h/w. The VPAV and MPAV are negatively correlated with ABSI, SBP, and DBP (*p* = 0.006–*p* < 0.001). Furthermore, we found a positive correlation between ABSI and blood pressure (i.e., SBP and DBP) (*p* < 0.001).

### 3.5. The SEM and Path Analysis

Table 4 shows the standardised path coefficients for the pathways of the proposed variables in cardiovascular diseases in structural equation modelling (SEM). A body shape index and systolic blood pressure were directly and significantly positively associated with the CVD; and ABSI has the largest β-coefficient of all the observed variables (i.e., β = 0.049). Two variables—food consumption score and vigorous PA volume—were all directly and significantly negatively associated with CVD (*p* = 0.004–*p* < 0.001). Food consumption scores and physical activity (i.e., MPAV and VPAV) were significantly and indirectly negatively associated with CVD.

ABSI was the only indirect significant positive factor associated with CVD. ABSI was also the only variable that was directly positively associated with blood pressures (i.e., SBP and DBP). Three variables—FCS, MPAV, and VPAV—were directly significantly negatively associated with SBP. Further, two variables—FCS and VPAV—were both directly and significantly negatively associated with DBP (*p* < 0.001). Food consumption score was indirectly significantly and positively associated with physical activity (i.e., MPAV and VPAV). On the other hand, ABSI was directly significantly and negatively associated with MPAV or VPAV. Moreover, FCS was the only variable that was directly and significantly negatively associated with ABSI (*p* < 0.001). Table S1 shows the un-standardised coefficient of direct–indirect effects and total effects of proposed variables on cardiovascular diseases in SEM.

The hypothesised model showed the overall model fit as SRMR 0.005, CFI 1.000, TLI 1.000, and RMSEA 0.000. We removed the non-significant pathways to increase the parsimony and then added a few additional paths to improve the model fitness. In particular, we dropped the non-significant correlation arrows (i.e., FCS → VPAV, FCS → MPAV, and DBP → CVD) and added the following correlations (i.e., SBP and DBP, and MPAV and VPAV). Thus, the final model had excellent fit, as shown from the values of SRMR 0.011, CFI 0.997, TLI 0.992, and RMSEA 0.019. Figure 1 shows the final model of the SEM.

## 4. Discussion

The present study examined the correlations between food insecurity, obesity, physical activity, and hypertension, as well as the potential to affect the rates of the incidence of cardiovascular disease in Indonesian adults. As hypothesised, the correlation test confirmed the positive correlations between ABSI and blood pressure (i.e., SBP and DBP) and/or CVD. Further, the correlation test and GEE analyses confirmed the negative correlations between FCS and the dependent variables (i.e., ABSI, SBP, DBP, and CVD). Furthermore, the correlation test and GEE analyses confirmed the negative correlations between physical activity (i.e., MPAV and VPAV) and the dependent variables (i.e., ABSI, SBP, DBP, and CVD).

### 4.1. Pathways from FCS to ABSI

Food-insecure people are more likely to be obese [34,35]. The former studies reported that a low-cost, poor-nutrition, high-energy-dense diet is linked to an increased waist circumference, mean of body mass index, and ABSI in food-insecure adults [11,36,37]. Therefore, the results from high-energy-dense and nutritionally poor diets are possible mechanisms to explain why low FCS is positively associated with the increase in ABSI. The result in our study was confirmed the possible mechanisms from the former studies that shown an increased one unit of FCS will decrease 0.057 unit of ABSI.

### 4.2. Pathways from ABSI to MPAV and VPAV

Obesity is negatively associated with physical activity [38,39,40,41]. Barning and colleagues observed the causal contributions of (abdominal) obesity to physical activity in adults [42]. Their study suggested an increase in adiposity level lead to physical activity decrease, but not vice versa. The result in our study was in line with former studies that body shape index is negatively associated with the moderate and/or vigorous physical activity volume.

### 4.3. Pathways from FCS to SBP and DBP

One of the food security indicators, the food consumption score, has been linked to hypertension in adults [1,33,43]. The clinically evident result from our study is that food insecurity is associated with hypertension (i.e., systolic and diastolic blood pressures), which is in line with the prior findings. Food-insecure people tend to replace a balanced diet of a variety of foods with a high-calorie diet [3,36] that leads to obesity. Obese people are more likely to have an increase in blood pressure [44,45]. Therefore, a hypothesis of a body shape-mediated direct and indirect pathway between FCS and SBP or DBP can be one of the mechanisms to bridge the gap between food security and hypertension [2,11]. The GEE test and SEM analysis in our study confirmed the effect of food security on SBP and/or DBP is mediated by ABSI.

Unbalanced meals lead to having a higher adiposity level, with low physical activity prone to increasing the possibility of getting hypertension and CVD [38,46,47,48,49,50,51]. Mathiassen and Hollema’s study can possibly also explain the role of food insecurity and hypertension [13]. Their study suggested that people with higher physical activity levels require a higher energy intake, but they often sacrifice food diversity for food quantity from a high-density diet. Further, PA level was defined by occupational physical activity (OPA) rather than by leisure time physical activity (LTPA; e.g., sports). However, food insecurity was not associated with physical activity when using the PA level definition from the LTPA [8,9,10,52].

The LTPA is more likely to be equal to moderate-to-vigorous PA that may be affected by the adiposity level. Prior studies suggested that higher adiposity leads to lower physical activity [42,52,53]. The physical activity volume in our study shows that the moderate physical activity volume (MPAV) is more likely to be equal to the OPA, meaning that food insecure people are more likely to have a low-to-moderate intensity of PA [52,54]. On the other hand, the vigorous physical activity volume (VPAV) in our study is more likely to be equal to the LTPA. Therefore, the food consumption score is positively correlated with the MPAV and negatively correlated with the VPAV, which is consistent with the former studies’ results. Further, ABSI, MPAV and VPAV are mediating pathways between FCS and SBP. On the other hand, ABSI and VPAV are mediating pathways between FCS and DBP.

### 4.4. Pathways from FCS to CVD

We observed the directly and significantly negative correlation between food consumption score (FCS) and VPAV with CVD. People who are food-secure have a better-quality, more diverse diet, so they have a higher food consumption score [1,11]. Food-secure individuals are less likely to be overweight, possibly because they were able to fulfil the need for food and physical activity to support their healthy lifestyle. For example, food-insecure individuals might have less ability to choose a balanced meal. The possible reason for this is that they often have high occupational activity, and their balanced meal would be replaced by the high calories diet. A concern is negative relationships, such as for food-insecure individuals in Indonesia often preferring less expensive, energy-dense, nutrient-poor foods, which can lead to obesity as well as a lack of LTPA [55,56].

Some investigators have suggested that the mediator of poor health outcome is physical activity [9]. Individuals who met the guideline recommended, which is a minimum level of physical activity (150 min/w or 10 METs h/w), have a 6% lower risk of hypertension, which reaches 12% or 33% at higher doses of LTPA [22]. We discovered the hypothesis that food-insecure individuals tend to consume high levels of calories, which leads to obesity and hypertension. Moreover, food-insecure individuals also often have high occupational activity, with or without obesity, and are less likely to perform LTPA, which leads to hypertension and the CVD incident. Prior studies also suggested that, compared to the DBP, SBP performs as a stronger predictor of CVD risk [57,58,59]. This hypothesis is possibly one of the mechanisms to explain that the pathways between FCS and CVD are mediated by ABSI, VPAV, and SBP [52].

The SEM has both strengths and limitations. The result of our earlier study allowed us to examine a range of modifiable variables in our theoretical model with potential influences on CVD incidence. Some certain limitations in this study included the fact that, among the numerous food security measurements, we used FFQ-based values as an indicator, which cannot deeply describe the food intake [60]. However, this method has been used and was adequately used to capture the food frequency and diversity of the diet [7,61]. Our study samples were restricted to a majority of the western part of Indonesia, so our findings cannot be generalised to some disadvantaged demographic locations. However, the characteristics of the sample population adequately represented the Indonesian demographic. Nevertheless, the negative relationships between FCS, VPAV, and CVD among these adults along with the positive relationship between ABSI and CVD have highlighted the importance of including food security in CVD prevention [45,48]. The direct and indirect effects in the model must be interpreted cautiously to imply the biological direction of the correlations because the model states causal assumptions and non-validated conclusions.

## 5. Conclusions

Further work is required to determine the in-depth causality among the variables. Of the multiple factors influencing the incidence of CVD, the modifiable risk factors were FCS, ABSI, and VPAV. Hence, the recommendations for CVD prevention should include targeting food insecurity, body shape index, and vigorous physical activity besides the measurement of blood pressure.

## Figures and Tables

**Table 1:** Characteristic of study participants.

Variable	All (*N* = 3955)		
	2007	2014	*p* Value ^a^
Age (years), mean	47(5)	54(5)	<0.001
Gender, %			
Women	2056(51.98)	2056(51.98)	
Men	1899(48.02)	1899(48.02)	
Education level, %			0.614
Low (<12 years)	3041(76.89)	3022(76.41)	
High (≥12 years)	914(23.11)	933(23.59)	
Marital status, %			0.182
Ever/Married	3900(98.61)	3913(98.94)	
Single/Never married	55(1.39)	42(1.06)	
Geographical residence, %			<0.001
Rural	1960(49.56)	1651(41.74)	
Urban	1995(50.44)	2304(58.26)	
Smoking habit, %			0.021
No smoking	2482(62.76)	2382(60.23)	
Currently smoking	1356(34.29)	1291(32.64)	
Former smoking	117(2.96)	282(7.13)	
Food consumption score, mean	52.02(22.76)	34.81(15.77)	<0.001
Food consumption group, %			<0.001
Poor	330(8.34)	815(20.61)	
Borderline	650(16.43)	1278(32.31)	
Acceptable	2975(75.22)	1862(47.08)	
Vigorous physical activity volume (MET h/w), mean	37.22(82.47)	22.02(65.63)	<0.001
Moderate physical activity volume (MET h/w), mean	36.47(48.80)	20.19(38.82)	<0.001
Using cholesterol medication, %			<0.001
No	3955(100)	3875(97.98)	
Yes	0(0)	80(2.02)	
Using diabetes medication, %			<0.001
No	3955(100)	3879(98.08)	
Yes	0(0)	76(1.92)	
Using hypertension medication, %			<0.001
No	3955(100)	3722(94.11)	
Yes	0(0)	233(5.89)	
Abdominal obesity ^b^, %			<0.001
No	2328(58.86)	1875(47.41)	
Yes	1627(41.14)	2080(52.59)	
Overweight ^c^, %			<0.001
No	2575(65.11)	2324(58.76)	
Yes	1380(34.89)	1631(41.24)	
WC (cm), mean	82.60(10.23)	86.11(10.75)	<0.001
BMI (kg/m^2^), mean	23.95(3.96)	24.57(3.90)	<0.001
ABSI (m^11/6^ kg^−2/3^), mean	0.0801(0.0062)	0.0821(0.0056)	<0.001
SBP (mmHg), mean	135(21)	143(25)	<0.001
DBP (mmHg), mean	83(12)	85(14)	<0.001
Hypertension ^d^, %			<0.001
No	2392(60.48)	1905(48.17)	
Yes	1563(39.52)	2050(51.83)	
Heart disease, %			<0.001
No	3955(100)	3874(97.95)	
Yes	0(0)	81(2.05)	
Stroke, %			<0.001
No	3955(100)	3919(99.09)	
Yes	0(0)	36(0.91)	
Cardiovascular disease ^e^, %			<0.001
No	3955(100)	3841(97.12)	
Yes	0(0)	114(2.88)	
Diabetes, %			<0.001
No	3955(100)	3789(95.80)	
Yes	0(0)	166(4.20)	

Abbreviations: MET h/w, metabolic equivalent of task hour per week; SD, standard deviation; WC, waist circumference; BMI, body mass index; ABSI, a body shape index; SBP, systolic blood pressure; DBP, diastolic blood pressure. Continuous data are presented as mean (SD) and categorical data are presented as n (%). ^a^ *t*-test or Chi Square test was used to compare between years 2007 and 2014 with significance *p*-value < 0.05; ^b^ Abdominal obesity was defined as being >80 cm for women or >90 cm for men; ^c^ Overweight was defined if the BMI was ≥25.1 kg/m^2^ and non-overweight if the BMI was <25.1 kg/m^2^; ^d^ Hypertension was defined as SBP ≥140 mmHg or DBP ≥90 mmHg; ^e^ Cardiovascular disease was defined when a participant had ever experienced one or more heart diseases and/or stroke.

**Table 2:** Correlation matrix of proposed variables on cardiovascular disease.

	FCS ^a^	ABSI	VPAV	MPAV	SBP	DBP	CVD
**FCS ^a^**	1.000						
**ABSI**	−0.057 **	1.000					
**VPAV**	−0.003	−0.114 **	1.000				
**MPAV**	0.058 **	−0.075 **	0.224 **	1.000			
**SBP**	−0.123 **	0.127 **	−0.075 **	−0.067 **	1.000		
**DBP**	−0.045 **	0.078 **	−0.084 **	−0.043 **	0.767 **	1.000	
**CVD**	−0.049 **	0.036 *	−0.036 *	−0.027 *	0.059 *	0.042 **	1.000

Abbreviations: FCS, food consumption score; ABSI, a body shape index; VPAV, vigorous physical activity volume; MPAV, moderate physical activity volume; SBP, systolic blood pressure; DBP, diastolic blood pressure; CVD, cardiovascular disease. ^a^ Food consumption score was used as the continuous data. * *p* < 0.05; ** *p* < 0.001.

**Table 3:** General estimating equations (GEE) result between food consumption score, blood pressures, and physical activity.

DV	IV	Unadjusted			Model 1			Model 2			Model 3		
		β Coef.	CI	*p*-Value	β Coef.	CI	*p*-Value	β Coef.	CI	*p*-Value	β Coef.	CI	*p*-Value
**SBP**	**FCS ^a^**	−0.13	(−0.16, −0.11)	<0.001	−0.08	(−0.10, −0.05)	<0.001	−0.08	(−0.10, −0.05)	<0.001	−0.08	(−0.11, −0.06)	<0.001
	**ABSI**	497.29	(411.49, 583.09)	<0.001	263.16	(176.24, 350.08)	<0.001	263.30	(176.37, 350.23)	<0.001	261.95	(175.08, 348.82)	<0.001
	**VPAV**	−2.34 × 10^−2^	(−3.03 × 10^−2^, −1.65 × 10^−2^)	<0.001	−1.31 × 10^−2^	(−2.00 × 10^−2^, −6.17 × 10^−3^)	<0.001	−1.35 × 10^−2^	(−2.05 × 10^−2^, −6.49 × 10^−3^)	<0.001	−1.30 × 10^−2^	(−2.00 × 10^−2^, −5.96 × 10^−3^)	<0.001
	**MPAV**	−3.53 × 10^−2^	(−4.68 × 10^−2^, −2.38 × 10^−2^)	<0.001	−1.58 × 10^−2^	(−2.72 × 10^−2^, −4.50 × 10^−3^)	0.006	−1.60 × 10^−2^	(−2.73 × 10^−2^, −4.60 × 10^−3^)	0.006	−1.62 × 10^−2^	(−2.76 × 10^−2^, −4.85 × 10^−3^)	0.005
**DBP**	**FCS ^a^**	−0.03	(−0.04, −0.01)	<0.001	−0.02	(−0.03, −0.01)	0.003	−0.03	(−0.04, −0.01)	<0.001	−0.03	(−0.04, −0.01)	<0.001
	**ABSI**	168.26	(120.76, 215.76)	<0.001	143.27	(93.99, 192.55)	<0.001	141.44	(92.23, 190.64)	<0.001	140.67	(91.50, 189.85)	<0.001
	**VPAV**	−1.45 × 10^−2^	(−1.83 × 10^−2^, −1.07 × 10^−2^)	<0.001	−1.31 × 10^−2^	(−1.70 × 10^−2^, −9.19 × 10^−3^)	<0.001	−1.18 × 10^−2^	(−1.58 × 10^−2^, −7.83 × 10^−3^)	<0.001	−1.15 × 10^−2^	(−1.55 × 10^−2^, −7.54 × 10^−3^)	<0.001
	**MPAV**	−1.25 × 10^−2^	(−1.89 × 10^−2^, −6.19 × 10^−3^)	<0.001	−1.03 × 10^−2^	(−1.67 × 10^−2^, −3.86 × 10^−3^)	0.002	−9.21 × 10^−3^	(−1.56 × 10^−2^, −2.79 × 10^−3^)	0.005	−9.35 × 10^−3^	(−1.58 × 10^−2^, −2.93 × 10^−3^)	0.004
**VPAV**	**FCS ^a^**	−0.01	(−0.09, 0.07)	0.779	−0.10	(−0.18, −0.03)	0.008	−0.01	(−0.09, 0.07)	0.800	−3.47 × 10^−3^	(−8.05 × 10^−2^, 7.36 × 10^−2^)	0.930
	**ABSI**	−1430.28	(−1704.20, −1156.36)	<0.001	−877.46	(−1154.20, −600.72)	<0.001	−848.37	(−1121.64, −575.10)	<0.001	−843.14	(−1116.13, −570.14)	<0.001
**MPAV**	**FCS ^a^**	0.12	(0.08, 0.17)	<0.001	4.90 × 10^−2^	(2.06 × 10^−3^, 9.59 × 10^−2^)	0.041	0.08	(0.03, 0.12)	0.002	0.07	(0.03, 0.12)	0.002
	**ABSI**	−560.45	(−725.02, −395.88)	<0.001	−277.39	(−446.58, −108.20)	0.001	−269.95	(−438.78, −101.11)	0.002	−270.84	(−439.67, −102.01)	0.002

Abbreviations: DV, dependent variable; IV, independent variable; β coef., β coefficient; CI, confidence intervals; SBP, systolic blood pressure; DBP, diastolic blood pressure; FCS, food consumption score; ABSI, a body shape index; VPAV, vigorous physical activity volume; MPAV, moderate physical activity volume. The generalised estimating equation (GEE) test was used with family (gaussian), link (identity), and correlation (independent). ^a^ Food consumption score was used as continuous data. Model 1 was adjusted for age and sex. Model 2 was adjusted for age, sex, and education level. Model 3 was adjusted for age, sex, education level, and smoking habit.

**Table 4:** Standardised path coefficients for pathways from food consumption score, body shape index, physical activity, blood pressures, and cardiovascular diseases in final model of SEM.

Pathway	Model for Cardiovascular Diseases				
	Coef.	SE	z	*p* Value	CI
**Direct**					
ABSI ← FCS	−0.057	0.011	−5.13	<0.001	(−0.079, −0.035)
MPAV ← ABSI	−0.075	0.011	−6.69	<0.001	(−0.097, −0.053)
VPAV ← ABSI	−0.114	0.011	−10.3	<0.001	(−0.136, −0.093)
**Indirect**					
SBP ←					
ABSI	0.112	0.011	10.09	<0.001	(0.090, 0.133)
FCS	−0.115	0.011	−10.5	<0.001	(−0.137, −0.094)
MPAV	−0.025	0.007	−3.46	0.001	(−0.040, −0.011)
VPAV	−0.057	0.011	−5.05	<0.001	(−0.079, −0.035)
DBP ←					
ABSI	0.067	0.011	5.94	<0.001	(0.045, 0.089)
FCS	−0.041	0.011	−3.71	<0.001	(−0.063, −0.020)
VPAV	−0.077	0.011	−6.83	<0.001	(−0.098, −0.055)
CVD ←					
ABSI	0.024	0.011	2.12	0.034	(0.002, 0.046)
FCS	−0.042	0.011	−3.73	<0.001	(−0.064, −0.020)
SBP	0.049	0.011	4.29	<0.001	(0.026, 0.071)
VPAV	−0.030	0.011	−2.66	0.008	(−0.052, −0.008)
**Correlation**					
SBP ← DBP	0.766	0.005	165.1	<0.001	(0.757, 0.775)
VPAV ← MPAV	0.218	0.011	20.34	<0.001	(0.197, 0.239)
**Model fitness**					
Chi-square test of model fit (*p* value)	<0.001				
CFI	0.997				
TLI	0.992				
SRMR	0.011				
RMSEA	0.019				

Abbreviations: Coef., coefficient; SE, standard error; CI, confidence interval; ABSI, a body shape index; FCS, food consumption score; MPAV, moderate physical activity volume; VPAV, vigorous physical activity volume; SBP, systolic blood pressure; DBP, diastolic blood pressure; CFI, comparative fit index; TLI, Tucker-Lewis index; SRMR, standardised root mean squared residual; RMSEA, root mean squared error of approximation. Structural equation modelling (SEM) test was used an independent variable (2007 and 2014) and a dependent variable (2007 and 2014). FCS, ABSI, MPAV, VPAV, SBP, and DBP were used as continuous data.

## Supplementary Materials

The following are available online at http://www.mdpi.com/1660-4601/15/8/1567/s1, Table S1: Direct and indirect effects of SEM variables on the cardiovascular diseases.
