# Supplementary material for: Pathways from Food Consumption Score to Cardiovascular Disease: A Seven-Year Follow-Up Study of Indonesian Adults
Source: Int J Environ Res Public Health. 2018 Jul 24;15(8):1567. doi: 10.3390/ijerph15081567 (PMC6121947; doi:10.3390/ijerph15081567)
Supplement: Supplementary file 1 [file ijerph-15-01567-s001.pdf]

International Journal of
Environmental Research and Public Health

MDPI

Supplementary Table 1. Direct and indirect effects of SEM variables on the cardiovascular diseases

DV	IV	β coefficient			*p-value*
		Direct	Indirect	Total	
**CVD**					
	**FCS**	-2.35×10^{-4} **	-4.18×10^{-5} **	-2.77×10^{-4}	< 0.001
	**ABSI**	0.48 *	0.19 **	0.66	0.003
	**VPAV**	-4.79×10^{-5} **	-4.14×10^{-6} **	-5.20×10^{-5}	0.004
	**MPAV**		-5.25×10^{-6} **	-5.25×10^{-6}	< 0.001
	**SBP**	2.47×10^{-4} **		2.47×10^{-4}	< 0.001
**SBP**					
	**FCS**	-1.25×10^{-1} **	-7.58×10^{-3} **	-1.33×10^{-1}	< 0.001
	**ABSI**	435.75**	35.81 **	471.56	0.003
	**VPAV**	-1.67×10^{-2} **		-1.67×10^{-2}	0.004
	**MPAV**	-2.12×10^{-2} **		-2.12×10^{-2}	< 0.001
**DBP**					
	**FCS**	-2.51×10^{-2} **	-2.62×10^{-3} **	-2.77×10^{-2}	< 0.001
	**ABSI**	144.21**	18.91 **	163.12	< 0.001
	**VPAV**	-1.32×10^{-2} **		-1.32×10^{-2}	< 0.001
**MPAV**					
	**FCS**		9.01×10^{-3} **	9.01×10^{-3}	< 0.001
	**ABSI**	-560.45		-560.45	0.003
**VPAV**					
	**FCS**		2.30×10^{-2} **	-2.30×10^{-2}	< 0.001
	**ABSI**	-1430.28 **		-1430.28	0.003
**ABSI**					
	**FCS**	-1.61×10^{-5} **		-1.61×10^{-5}	< 0.001

Abbreviations: DV, dependent variable; IV, independent variable; CVD, cardiovascular diseases; ABSI, a body shape index; FCS, food consumption score; MPAV, moderate physical activity volume; VPAV, vigorous physical activity volume; SBP, systolic blood pressure; DBP, diastolic blood pressure. * $p < 0.05$; ** $p < 0.001$

Supplementary file: Model description

Our model was constructed by linking the modifiable variables including food insecurity indicator (i.e. food consumption score) with the obesity indicator (body shape index), the hypertension indicators (i.e. SBP, DBP), the physical activity (i.e. VPAV, MPAV), and the outcome health (i.e. CVD). The first dimension measured in our hypothetical model was a body shape index as the mediator variable between the food consumption score and hypertension. This first hypothetical was done in our previous work. The food consumption score was associated, and mediated some part, with the body shape index, and hypertension (i.e. SBP, DBP) [1]. These findings were in line with the previous studies that suggested consumption of high energy dense and poorly nutrient diet associated with low food consumption score (FCS). The high-energy intake leads to the increase of overweightness, and higher prevalence of chronic disease (e.g. hypertension, diabetes, and cardiovascular disease) [2-5].

Further, the cardiovascular disease main risk factors are hypertension, obesity, high blood sugar, and high low-density lipoprotein. The limited data in our study that the Indonesian Family Life Survey do not provide the blood sugar (i.e. fasting plasma glucose) and the low-density lipoprotein (LDL) values. Therefore, we used the blood pressures and body shape index as the indicator of hypertension and obesity, respectively. The final aspect measured in our hypothetical model was the effect of physical activity on the relationship between food insecurity and chronic diseases. The physical activity together with the diet are basic factors associated with the obesity [6-11]. In contrast, the physical activity is not associated with the food insecurity [11-13] and suggested that the increased adiposity level causes in a reduction in physical activity [7]. To determine where the physical activity can counterbalance the risk associated with food insecurity and CVD, we used the SEM to explore the complex direct and indirect factor variables. Hence, we hypothesized our SEM model with the proposing variables, such as food consumption score, body shape index, physical activity, hypertension on cardiovascular disease.

References

1. Isaura, E.R.; Chen, Y.C.; Yang, S.H. The association of food consumption scores, body shape index, and hypertension in a seven-year follow-up among indonesian adults: A longitudinal study. *Int J Environ Res Public Health* **2018**, *15*, 175.
2. Franklin, B.; Jones, A.; Love, D.; Puckett, S.; Macklin, J.; White-Means, S. Exploring mediators of food insecurity and obesity: A review of recent literature. *J Community Health* **2012**, *37*, 253-264.
3. Drewnowski, A.; Specter, S.E. Poverty and obesity: The role of energy density and energy costs. *Am J Clin Nutr* **2004**, *79*, 6-16.
4. Laraia, B.A. Food insecurity and chronic disease. *Adv Nutr* **2013**, *4*, 203-212.
5. Seligman, H.K.; Laraia, B.A.; Kushel, M.B. Food insecurity is associated with chronic disease among low-income nhanes participants. *J Nutr* **2010**, *140*, 304-310.
6. Gunter, K.B.; Jackson, J.; Tomayko, E.J.; John, D.H. Food insecurity and physical activity insecurity among rural oregon families. *Prev Med Rep* **2017**, *8*, 38-41.

7. Barning, F.; Abarin, T. Assessing the causality factors in the association between (abdominal) obesity and physical activity among the newfoundland population-a mendelian randomization analysis. *Genet Epigenet* **2016**, *8*, 15-24.
8. Brown, T.; Smith, S.; Bhopal, R.; Kasim, A.; Summerbell, C. Diet and physical activity interventions to prevent or treat obesity in south asian children and adults: A systematic review and meta-analysis. *Int J Environ Res Public Health* **2015**, *12*, 566-594.
9. Choi, J.E.; Ainsworth, B.E. Associations of food consumption, serum vitamins and metabolic syndrome risk with physical activity level in middle-aged adults: The national health and nutrition examination survey (nhanes) 2005-2006. *Public Health Nutr* **2016**, *19*, 1674-1683.
10. Dhana, K. Obesity, physical activity, and cardio-metabolic disorders in older adults. **2016**.
11. Seligman, H.K.; Bindman, A.B.; Vittinghoff, E.; Kanaya, A.M.; Kushel, M.B. Food insecurity is associated with diabetes mellitus: Results from the national health examination and nutrition examination survey (nhanes) 1999-2002. *J Gen Intern Med* **2007**, *22*, 1018-1023.
12. Seligman, H.K.; Schillinger, D. Hunger and socioeconomic disparities in chronic disease. *N Engl J Med* **2010**, *363*, 6-9.
13. Berkowitz, S.A.; Baggett, T.P.; Wexler, D.J.; Huskey, K.W.; Wee, C.C. Food insecurity and metabolic control among u.S. Adults with diabetes. *Diabetes Care* **2013**, *36*, 3093-3099.

© 2018 by the authors. Submitted for possible open access publication under the terms and conditions of the Creative Commons Attribution (CC BY) license (http://creativecommons.org/licenses/by/4.0/).
